# Long-term effect of transcatheter mitral valve edge-to-edge repair on left ventricular function and mitral regurgitation severity: a single-center experience

**DOI:** 10.1186/s43044-025-00684-6

**Published:** 2025-09-08

**Authors:** Jamilah AlRahimi, Wasan Alghamdi, Raghad Alatabani, Refal Almuzil, Reemas Alahmadi, Amjad SaemAldahar, Alhanouf Alotaibi, Fatima Ahmed, Yasser Ismail, Ibrahim Jelaidan

**Affiliations:** 1 Department of Cardiology, King Abdulaziz Medical City, Ministry of National Guard Health Affairs, King Abdullah International Medical Research Center, Jeddah, Saudi Arabia; 2 Collage of Medicine, King Saud bin Abdulaziz University for Health Sciences, Jeddah, Saudi Arabia; 3 King Abdullah International Medical Research Center, Jeddah, Saudi Arabia; 4 Collage of Applied Medical Sciences, King Saud bin Abdulaziz University for Health Sciences, Jeddah, Saudi Arabia; 5 King Abdulaziz Medical City, Ministry of National Guard Health Affairs, Jeddah, Saudi Arabia

**Keywords:** Mitral regurgitation, MitraClip, Ejection fraction, Heart failure, Transcatheter edge-to-edge repair

## Abstract

**Background:**

Long-term outcomes of transcatheter mitral valve edge-to-edge repair (TEER) are compared with medical therapy remain under investigation. This study evaluated the 3-year effects of MitraClip on mitral regurgitation (MR) severity, ventricular remodeling, and clinical outcomes in high surgical-risk patients.

**Methods:**

A single-center retrospective cohort included 31 MitraClip patients (2016–2023) and 30 contemporaneous controls on maximally tolerated guideline-directed medical therapy. Anatomical suitability was based on EVEREST criteria. Echocardiography was performed at baseline, 1, 2, and 3 years. Outcomes included MR severity, left ventricular ejection fraction (LVEF), right-sided parameters, biomarkers, NYHA class, 6-min walk test, heart failure (HF) hospitalizations, and all-cause mortality.

**Results:**

Mean age was 63.2 ± 14.5 MitraClip vs. 72.1 ± 8.9 years controls, with secondary MR in 61.3% vs. 56.7%. Severe pulmonary hypertension (SPAP > 50 mmHg) was present in 25% of primary and 55% of secondary MR patients. No procedural mortality occurred. MR improved significantly with MitraClip (93.0% mild to moderate at 1st year, 83.9% at 3rd year) while controls remained severe (*p* < 0.001). LVEF remained stable (~ 33–35%, *p* = 0.412) without correlation to MR reduction. HF hospitalizations were lower with MitraClip (58.1%, 2.1 ± 1.8 admissions) vs. controls (7.6 ± 3.2, *p* < 0.001). Within MitraClip, secondary MR patients had higher rates (68.4% vs. 41.7%, *p* = 0.042). SPAP decreased after MitraClip but rose in controls (*p* = 0.015). BNP declined with MitraClip but increased in controls (*p* = 0.01). At 3 years, more MitraClip patients were in NYHA I/II (41.9% vs. 15%), 6MWT improved while controls declined, and mortality was lower (12.9% vs. 45%, *p* < 0.01).

**Conclusion:**

TEER provides durable MR reduction, BNP stabilization, improved functional capacity, and survival benefits compared with medical therapy in high-risk surgical patients. Primary MR patients might derive greater benefit than those with secondary MR. Larger multicenter studies are warranted.

## Introduction

Mitral regurgitation (MR) is among the most common valvular heart diseases, especially in older adults, and can lead to significant morbidity and mortality if not treated [1–3]. Surgical repair or replacement of the mitral valve remains the standard treatment for symptomatic severe MR; however, many patients are considered inoperable due to age, frailty, or other health issues [4]. For these high-risk patients, transcatheter mitral valve edge-to-edge repair (TEER) with the MitraClip (Abbott- CA- USA) device has become a less invasive option [5].

Clinical trials such as EVEREST, COAPT, and MITRA-FR have shown the safety and effectiveness of MitraClip in reducing MR severity and enhancing quality of life in carefully selected patients [6–8]. However, despite these promising results, the effect of TEER on left ventricular systolic function, especially in patients with impaired baseline ejection fraction (EF), remains not fully understood. Although reducing chronic volume overload should theoretically promote reverse remodeling, the presence of irreversible myocardial fibrosis or advanced cardiomyopathy may blunt functional recovery [9]. Additionally, MR etiology (primary vs. secondary) plays a crucial role in procedural response, as secondary MR is often proportionate to underlying left ventricular dysfunction, whereas primary MR represents a more valve-driven pathology.

Understanding the trajectory of left ventricular function after MR reduction is essential for optimizing patient selection, procedural timing, and long-term management strategies [9]. Anatomical assessment based on the EVEREST criteria remains the cornerstone of suitability evaluation, though real-world practice increasingly includes patients with more complex anatomies, such as leaflet calcification or restricted leaflet mobility, when feasible with current device iterations. Additionally, the pathophysiological differences between primary (degenerative) and secondary (functional) MR indicate that outcomes may differ significantly based on etiology. Real-world, longitudinal data are needed to better understand these differences and to guide tailored treatment approaches.

This study aims to evaluate the long-term impact of MitraClip therapy on MR severity and EF in a high surgical-risk cohort treated at a single tertiary cardiac center. By analyzing serial echocardiographic data over 3 years, we seek to describe the durability of MR reduction, trends in left ventricular function, and outcome differences based on MR etiology, while adding to the limited evidence from Middle Eastern populations.

## Methods

### Design and patients

This retrospective cohort study was conducted at a tertiary cardiac center in Saudi Arabia. The study included patients who underwent MitraClip implantation between 2016 and 2023. Among 44 patients treated during this period, 31 met the inclusion criteria of having complete transthoracic echocardiographic (TTE) follow-up at 1, 2, and 3 years. Thirteen patients were excluded due to incomplete TTE data or loss to follow-up. Despite a mean cohort age of 63 years, all patients were deemed inoperable or at prohibitive surgical risk by a multidisciplinary heart team based on advanced age, comorbid conditions, such as chronic kidney disease (CKD), chronic lung disease, and frailty, impaired ventricular function or elevated surgical risk scores quantified using the Society of Thoracic Surgeons (STS) Predicted Risk of Mortality and/or EuroSCORE II. A contemporaneous control group (n = 30) was identified, consisting of patients with severe symptomatic MR (both primary and secondary) evaluated during the same study period but deemed unsuitable for MitraClip implantation due to anatomical unsuitability, frailty, or prohibitive comorbidities. These patients received maximally tolerated guideline-directed medical therapy (GDMT) and standard heart failure management. Clinical and echocardiographic data were collected retrospectively over 3 years.

#### Anatomical selection criteria and procedural details

Anatomical suitability for TEER was assessed according to the EVEREST criteria, with transesophageal echocardiography (TEE) guiding initial evaluation. Procedures were performed under general anesthesia with fluoroscopic and TEE guidance. Device generation, number of clips implanted per patient, and procedural complications were systematically recorded.

#### Medical therapy

All patients received guideline-directed medical therapy (GDMT) tailored to MR etiology and heart failure status, including maximally tolerated doses of beta-blockers, ACE inhibitors/angiotensin receptor blockers (ACEi/ARB), or angiotensin receptor–neprilysin inhibitors (ARNI), mineralocorticoid receptor antagonists, and diuretics as needed. Sodium-glucose cotransporter-2 inhibitors (SGLT2i) were initiated in eligible patients in recent years. Blood pressure and heart rate control were maintained with beta-blockers and antihypertensives throughout follow-up, unless contraindicated. Cardiac resynchronization therapy (CRT) or implantable cardioverter-defibrillators (ICD) were used where indicated.

### Echocardiography

Baseline and follow-up transthoracic echocardiographic (TTE) studies were performed using the Philips iE33 system (Philips, Amsterdam, Netherlands). Quantitative assessments included left ventricular end-diastolic volume (LVEDV), end-systolic volume (LVESV), end-diastolic diameter (LVEDD), and end-systolic diameter (LVESD). Ejection fraction (EF) was calculated using Simpson’s biplane methods [10], whenever feasible; in patients with suboptimal acoustic windows, the Teichholz method was applied, an acknowledged limitation. Mitral regurgitation (MR) severity was assessed according to American Society of Echocardiography (ASE) guidelines [11], incorporating multiple parameters: color Doppler jet area, vena contracta, regurgitant volume (RVol), regurgitant fraction (RF), and effective regurgitant orifice area (EROA). PISA-derived EROA was not used after MitraClip implantation due to post-TEER geometric distortion. Pulmonary vein flow patterns and left atrial dimensions were also evaluated. Three dimensional quantification (3D echo) was not systematically available.

Right-sided parameters included RV basal diameter, tricuspid annular plane systolic excursion (TAPSE), tricuspid annular systolic velocity (RV S′), and systolic pulmonary artery pressure (SPAP), which was estimated from tricuspid regurgitant velocity and right atrial pressure (derived from IVC size and collapsibility.

Comprehensive echocardiographic follow-ups were scheduled at approximately 1, 2, and 3 years post-procedure. Intra-procedural trans esophageal echocardiography (TEE) was performed in all patients, and selective follow-up TEE (n = 5) was conducted for recurrent MR or inconclusive TTE findings, identifying mechanisms such as single-leaflet device attachment (SLDA), leaflet disease progression, or ventricular remodeling. These annual intervals were selected to align with evidence from major clinical trials such as COAPT and MITRA-FR and reflect common clinical practice in high-risk populations. All echocardiographic studies were independently reviewed by two experienced cardiologists who were blinded to clinical outcomes. For LVEF, discrepant values were resolved by averaging the two measurements. For MR parameters (EROA, vena contracta, regurgitant volume), discrepancies in interpretation were resolved by consensus discussion.

For the control cohort, echocardiographic measurements were obtained from routine clinically indicated studies at baseline and during follow-up, using the same acquisition and analysis standards as the MitraClip group.

### Data and outcomes

In addition to the echocardiographic data, demographics, comorbidities, and the etiology of mitral valve regurgitation were collected for both the MitraClip and control cohorts. Data were extracted from paper and electronic medical charts. The primary outcomes were all-cause mortality, and HF hospitalizations during follow-up. The secondary outcomes included changes in: (a) B-type natriuretic peptide (BNP) levels measured at baseline, 1 year, and 3 years. (b) **Functional capacity:** assessed by the 6-min walk test (6MWT) at baseline and follow-up, when available. (c) New York Heart Association (NYHA) functional class, at baseline and follow-up.

Mechanisms of recurrent MR and causes of death were also documented. Kaplan–Meier analysis was used to assess survival over 3 years. Echocardiographic data were interpreted alongside BNP levels, functional capacity (6MWT), and NYHA class to provide integrated assessment of hemodynamic and clinical outcomes.

### Ethical consideration

The study was approved by the local Institutional Review Board (IRB number: SP23J/123/08), and the need for patients’ consent was waived because of the retrospective design.

### Statistical analysis

Statistical analysis was performed using JMP software (SAS Institute, Cary, USA). Continuous variables were reported as mean ± standard deviation (SD) or median with interquartile range (IQR), as appropriate. The normality of the data was assessed visually using histograms and the Shapiro–Wilk test. Between-group comparisons (MitraClip vs. Control) were performed with Student’s t-test or Mann–Whitney U test for continuous variables, and Chi-square or Fisher’s exact test for categorical variables. Changes over time were analyzed using repeated-measures ANOVA or linear mixed-effects models, with group vs time interaction terms to compare trajectories between MitraClip and Control cohorts. Right-sided parameters (RV basal diameter, TAPSE, RV S′, and SPAP) were analyzed longitudinally.. BNP values were log-transformed; for controls, peak BNP values during HF hospitalizations were used for the 3-year analysis. Six-minute walk test (6MWT) distances and NYHA class were analyzed at baseline and follow-up; NYHA shifts were assessed with the McNemar test. Spearman’s rank correlation was used to assess relationships between MR and EF changes, as well as BNP and heart failure hospitalizations. All-cause mortality was analyzed using Kaplan–Meier survival analysis. Survival curves were compared between groups using the log-rank test. Patients with missing data were excluded, and complete case analysis was done. While the sample size was determined by the availability of patients with complete longitudinal data rather than formal power calculation, we conducted a post hoc power analysis to assess the study's ability to detect clinically meaningful differences. Based on previous studies reporting effect sizes for MR reduction following TEER [6, 7], our sample of 31 patients provided 80% power to detect a large effect size (Cohen's d ≥ 0.8) for within-subject changes in MR severity at α = 0.05. For between-group comparisons (primary vs. secondary MR), the study had limited power (< 50%) for moderate effect sizes (d = 0.5). A two-sided p-value of < 0.05 was considered statistically significant.

## Results

### Demographic and baseline clinical characteristics

Baseline clinical and echocardiographic characteristics were generally well balanced between the MitraClip (n = 31) and control groups (n = 30) (Table 1). The distribution of MR etiology, age, sex, and baseline LVEF did not differ significantly between groups. However, the control cohort demonstrated persistant NYHA Class III–IV and significantly higher pulmonary pressures, with a mean SPAP of **57.9 ± 9.0 mmHg** compared with **51.3 ± 9.0 mmHg** in the MitraClip group (p = 0.01). In contrast, baseline functional capacity as assessed by the 6-min walk test (6MWT) was slightly higher in controls (**290 ± 25 m** vs. **280 ± 30 m**, *p* = 0.12), though this difference was not statistically significant.

**Table 1:** Demographic and baseline clinical characteristics of mitraclip and control patients

Variable	MitraClip Cohort (n = 31)	Control Cohort (n = 30)
Age (years), mean ± SD	63.2 ± 14.5	72.1 ± 8.9
Male sex, n (%)	20 (64.5%)	16 (53.3%)
Diabetes mellitus, n (%)	18 (58%)	17 (56.7%)
Hypertension, n (%)	23 (74%)	22 (73.3%)
Coronary artery disease, n (%)	21 (68%)	20 (66.7%)
Chronic kidney disease, n (%)	10 (32%)	14 (46.7%)
Atrial fibrillation, n (%)	15 (48%)	9 (30%)
Baseline LVEF (%), mean ± SD	33.8 ± 11.3	34.6 ± 11.2
MR Etiology: Primary, n (%)	12 (38.7%)	13 (43.3%)
MR Etiology: Secondary, n (%)	19 (61.3%)	17 (56.7%)
Baseline NYHA Class III–IV (%)	~ 70%	90%
Baseline SPAP (mmHg, mean ± SD)	51.3 ± 9.0	57.9 ± 9.0
Baseline 6MWT (m, mean ± SD)	280 ± 30	290 ± 25

LVEF, left ventricular ejection fraction; MR, mitral regurgitation; SPAP, Systolic Pulmonary Artery Pressure, 6MWT, Six Minutes Walk Test, SD, standard deviation

The mean age of the MitraClip group was 63.2 ± 14.5 years, with a predominance of male patients (64.5%). Comorbidities were prevalent, including diabetes (58%), hypertension (74%), coronary artery disease (68%), chronic kidney disease (32%), and atrial fibrillation (48%). Corresponding values in the control group were diabetes (56.7%), hypertension (73.3%), coronary artery disease (66.7%), chronic kidney disease (30%), and atrial fibrillation (46.7%), with no significant between-group differences. The mean STS Predicted Risk of Mortality was 9.3 ± 3.2%. The baseline mean left ventricular EF was 33.8 ± 11.3% in the MitraClip cohort and 34.6 ± 11.2% in controls. Mitral regurgitation (MR) etiology was primary in 38.7% and secondary in 61.3% of MitraClip patients,, and primary in 43.3% and secondary in 56.7% of controls (Table 1).

### Procedural data

Complex anatomies including significant leaflet calcification, restricted posterior leaflet mobility, or eccentric jets were present in 4 patients (12.9%) but were considered feasible for TEER after Heart Team consensus. MitraClip NT and NTW devices (Abbott Vascular, Santa Clara, CA, USA) were used. Most patients (n = 25) received one clip; five received two clips, and one received three clips. No procedural mortality occurred. Procedural safety was high, with a low incidence of major complications, only two notable complications during the index hospitalization: one case of single-leaflet device attachment (SLDA) and one pericardial effusion requiring drainage. Both events were managed successfully without lasting sequelae. No device embolization, no significant leaflet injury, or major vascular access injury and no in-hospital mortality.

### Echocardiographic changes

At baseline, mean LVEF was 33.8 ± 11.3% [95% CI 30.3–37.3] in the MitraClip cohort and 34.6 ± 11.2% in the control cohort, with no significant between-group difference (*p* = 0.78). In MitraClip patients, LVEF showed no significant longitudinal changes across the follow-up period: 35.2 ± 11.9% at 1 year (*p* = 0.521), 33.7 ± 11.8% at 2 years (*p* = 0.674), and 32.8 ± 9.6% at 3 years (*p* = 0.412). In contrast, LVEF in the control group declined slightly but remained statistically nonsignificant over 3 years (34.6 ± 11.2% to 31.0 ± 12.5%, *p* = 0.09) (Table 2).

**Table 2:** Ejection fraction trends in mitraclip vs control patients

Timepoint	MitraClip (n = 31) Mean EF (% ± SD) [95% CI]	Control (n = 30) Mean EF (% ± SD) [95% CI]
Baseline	33.8 ± 11.3 [30.3–37.3]	**34.6 ± 11.2 [30.4–38.8]**
1 st Year Follow-up	35.2 ± 11.9 [31.6–38.8]	**33.5 ± 11.8 [29.2–37.8]**
2nd Year Follow-up	33.7 ± 11.8 [29.5–37.9]	**32.0 ± 12.1 [27.6–36.4]**
3rd Year Follow-up	32.8 ± 9.6 [28.2–37.4]	**31.0 ± 12.5 [26.4–35.6]**

CI, confidence interval; EF, ejection fraction; SD, standard deviation. No statistically significant differences were observed between groups at any time point (all *p* >0.05)

Stratification by EF categories (Severe < 30%, Moderate 30–39%, Mild 40–49%, Borderline 50–54%, and Normal ≥ 55%) demonstrated that most patients in both groups consistently remained within the moderate and severe EF ranges.

Left ventricular volumes demonstrated divergent remodeling patterns. In the MitraClip group, mean LVEDV declined from 151.4 ± 35.2 mL at baseline to 143.1 ± 37.5 mL at 3 years, and LVESV from 101.3 ± 28.7 mL to 95.5 ± 29.8 mL. Similarly, linear dimensions, LVEDD decreased from 5.8 ± 0.6 cm to 5.6 ± 0.6 cm and LVESD from 4.4 ± 0.5 cm to 4.2 ± 0.5 cm. These changes were insignificant (ANOVA *p* > 0.05). By contrast, controls demonstrated progressive LV dilatation, with LVEDV increasing from 160 ± 40 to 176 ± 46 mL, LVESV from 105 ± 30 to 122 ± 36 mL, and corresponding increases in LVEDD and LVESD (Table 3).

**Table 3:** Left ventricle volume parameters in mitraclip vs control patients

Timepoint	MitraClip (n = 31)				Control (n = 30)			
	Mean LVEDV (mL) ± SD	Mean LVESV (mL) ± SD	Mean LVEDD (cm) ± SD	Mean LVESD (cm) ± SD	Mean LVEDV (mL) ± SD	Mean LVESV (mL) ± SD	Mean LVEDD (cm) ± SD	Mean LVESD (cm) ± SD
Baseline	151.4 ± 35.2	101.3 ± 28.7	5.8 ± 0.6	4.4 ± 0.5	160 ± 40	105 ± 30	5.9 ± 0.6	4.5 ± 0.5
1 st Year Follow-up	148.7 ± 38.0	99.2 ± 31.1	5.7 ± 0.6	4.3 ± 0.5	165 ± 42	110 ± 32	6.0 ± 0.6	4.6 ± 0.5
2nd Year Follow-up	145.2 ± 36.8	97.4 ± 30.6	5.7 ± 0.6	4.3 ± 0.4	170 ± 44	116 ± 34	6.1 ± 0.6	4.7 ± 0.5
3rd Year Follow-up	143.1 ± 37.5	95.5 ± 29.8	5.6 ± 0.6	4.2 ± 0.5	176 ± 46	122 ± 36	6.2 ± 0.7	4.8 ± 0.6

LVEDV, left ventricular end-diastolic volume; LVESV, left ventricular end-systolic volume; LVEDD, left ventricular end-diastolic diameter; LVESD, left ventricular end-systolic diameter

^*^p-value (Group × Time) 0.02 (LVEDV) 0.03 (LVESV) 0.04 (LVEDD) 0.05 (LVESD)

**Fig. 1:**
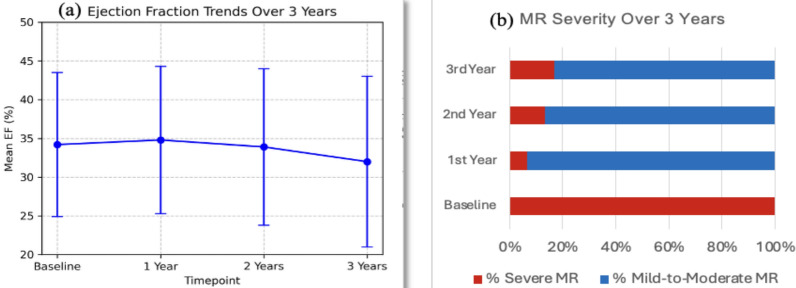
Longitudinal trends in ejection fraction (EF) and mitral regurgitation (MR) severity post-MitraClip. **a** EF trends over 3 years. **b** MR severity trends over 3 years

In contrast to EF, MR severity showed statistically significant improvement over time after MitraClip. At baseline, all patients had severe MR. By the first follow-up, 93.3% had improved to mild-to-moderate or better, and only 6.7% still had severe MR. This favorable pattern was maintained into the second year (86.6% mild-to-moderate or better) but showed some regression by year three, where 17% of patients had recurrent severe MR, due to SLDA (n = 1), progression of leaflet disease (n = 2), and adverse LV remodeling (n = 2). Overall,83.9% of MitraClip patients remained in the mild-to-moderate or better category at 3 years. MR grade reduction was significant (Friedman *p* < 0.001), and ordinal scoring (on a scale where 4 = Severe, 3 = Moderate to Severe, 2 = Moderate, 1 = Mild to Moderate, 0 = Mild) confirmed a consistent downward trend in MR grade across follow-ups (Table 4). Despite MR reduction, EF did not show parallel improvement (Fig. 1).

**Table 4:** Mitral regurgitation severity trends in mitraclip vs control patients

Timepoint	MitraClip (n = 31)		Control (n = 30)		p-value (Group × Time)
	Severe MR %	Mild-to-Moderate or Better MR %	Severe MR %	Mild-to-Moderate or Better MR %	
Baseline	100%	0	100%	0%	
1 st Year Follow-up	6.7%	93.3%	85%	15%	< 0.001
2nd Year Follow-up	13.4%	86.6%	88%	12%	< 0.001
3rd Year Follow-up	17%	83.9%	90%	10%	< 0.001

MR, mitral regurgitation

**Fig. 2:**
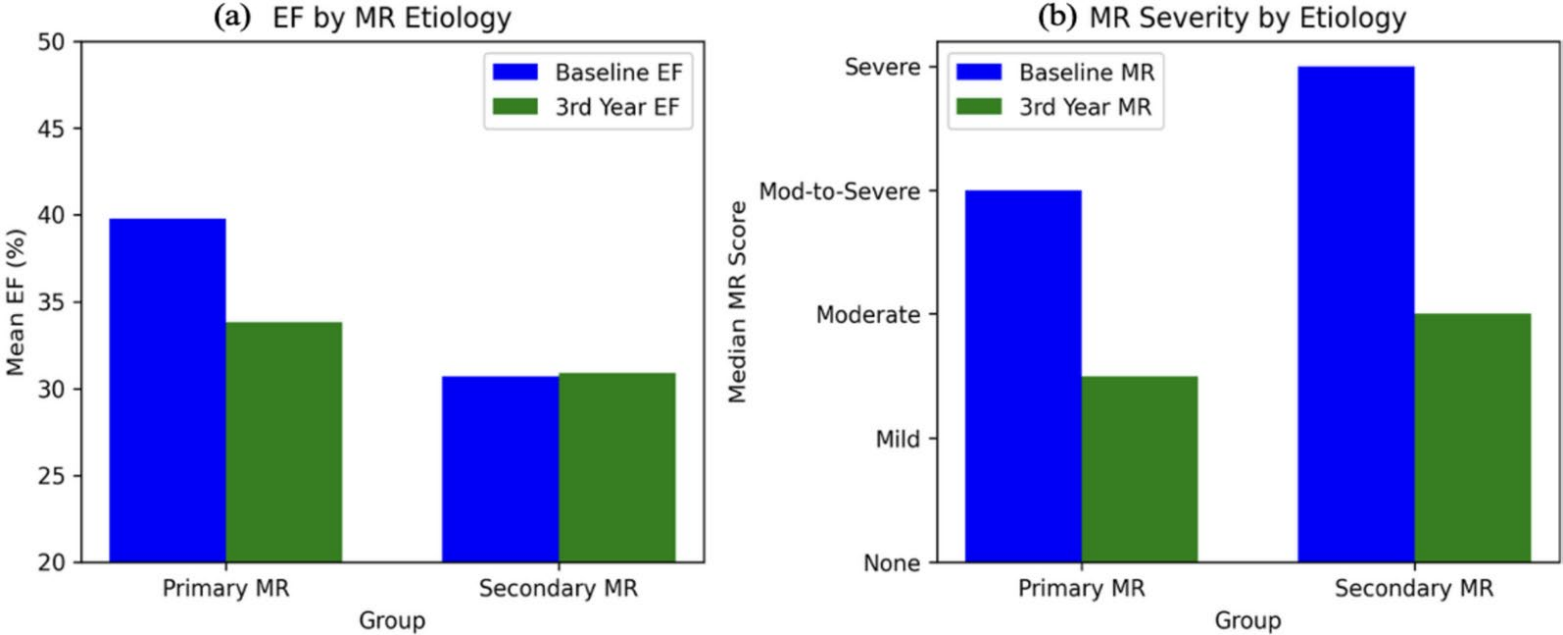
EF and MR outcomes by MR etiology. **a** Mean EF at baseline and 3-year follow-up **b** Median MR severity scores at baseline and 3-year follow-up

No correlation was observed between changes in MR severity and EF (Spearman ρ = 0.071, *p* = 0.735). Similarly, there were no statistically significant differences in changes between primary and secondary MR groups for LVEDV, LVESV, or EF within the MitraClip cohort. Patients with primary MR had a higher baseline EF (mean 42.3 ± 7.8%, 95% CI 37.5–47.1) and greater MR improvement (median score from 3 to 1.5) compared to those with secondary MR (median score from 4 to 2.0) (baseline EF 29.5 ± 10.2%, 95% CI 25.4–33.6; follow-up EF 31.2 ± 12.1%, 95% CI 26.3–36.1; MR score from 4 to 2.0) (Fig. 2). Table 5 presents detailed differences in left ventricular (LV) volumes and ejection fraction (EF) at baseline and at 3-year follow-up in patients with primary and secondary mitral regurgitation.

**Table 5:** LV volumes, ejection fraction (EF) and mitral regurgitation (MR) differences at baseline and 3 year in patients with primary and secondary mitral regurgitation

Group	LVEDV (mL ± SD)		LVESV (mL ± SD)		Mean EF (% ± SD)		MR (Median)	
	Baseline	3rd Year (mean change)	Baseline	3rd Year (mean change)	Baseline	3rd Year (mean change)	Baseline	3rd Year
Primary MR	145.8 ± 32.1	135.6 ± 30.5 (− 10.2 mL)	84.2 ± 21.5	79.1 ± 22.3 (− 5.1 mL)	42.3 ± 7.8% [95% CI 37.5–47.1]	38.7 ± 9.2% [95% CI 33.1–44.3]	3 Mod to-Severe	1.5 Mild-Mod
Secondary MR	155.2 ± 36.5	147.9 ± 39.1 (− 7.3 mL)	112.8 ± 26.7	106.4 ± 28.5 (− 6.4 mL)	29.5 ± 10.2% [95% CI 25.4–33.6]	31.2 ± 12.1% [95% CI 26.3–36.1]	4 Severe	2.0 Moderate

CI, confidence interval; EF, ejection fraction; SD: standard deviation

In contrast, controls with primary or secondary MR demonstrated persistent severe MR and progressive LV dilatation without meaningful improvement in EF. Control primary MR patients maintained modestly higher EF at baseline (~ 38–40%) but experienced progressive decline with recurrent HF admissions, while secondary MR controls consistently showed the lowest EF (~ 28–30%) and the largest LV volumes. These findings reinforce that the differential remodeling patterns observed after MitraClip were specific to the intervention, whereas controls followed the natural trajectory of MR with adverse LV remodeling and persistent hemodynamic burden.

### Transmitral gradients

The transmitral mean gradient increased modestly after MitraClip implantation, rising from 2.6 ± 0.9 mmHg at baseline to 4.5 ± 2.6 mmHg at 3 years (Δ ≈ + 2.0 mmHg; *p* = 0.018; n = 12). Most patients maintained gradients within the physiologic range of 2–6 mmHg, with the highest value observed being 7.2 mmHg in a single patient who received two clips. In contrast, gradients in the control group remained unchanged over follow-up, averaging 2.4 ± 0.8 mmHg at baseline and 2.6 ± 0.9 mmHg at 3 years (*p* = 0.41). No control patients developed clinically significant mitral stenosis. The overall group × time effect confirmed that the modest rise in transmitral gradients was specific to MitraClip patients (*p* = 0.02), though values generally remained well below the threshold associated with functional mitral stenosis.

### Right-sided hemodynamics

Right-sided parameters demonstrated favorable changes in MitraClip patients compared with controls. In the MitraClip group, severe pulmonary hypertension, systolic pulmonary artery pressure (SPAP) > 50 mmHg, was present in 25% of primary and 55% of secondary MR patients. SPAP declined from 44 ± 7 to 39.4 ± 7 mmHg in primary MR and from 56 ± 9 to 48.8 ± 9 mmHg in secondary MR. By contrast, controls experienced a mild but nonsignificant rise in SPAP (Primary: 50 ± 8 → 52 ± 8 mmHg; Secondary: 64 ± 9 → 66 ± 9 mmHg) (Table 6).

**Table 6:** Right-sided echocardiographic parameters at baseline and follow-up according to MR etiology and groups

Group/Time	RV Basal Diameter (cm)	TAPSE (mm)	RV S′ (cm/s)	SPAP (mmHg)
	Baseline → 3-Year	Baseline → 3-Year	Baseline → 3-Year	Baseline → 3-Year
MitraClip				
Primary MR (n = 12)	3.56 ± 0.10 → 3.61 ± 0.08 (*p* = 0.07)	20.3 ± 0.5 → 20.5 ± 1.2 (*p* = 0.02)	10.6 ± 0.2 → 10.6 ± 0.5 (*p* = 0.03)	44 ± 7 → 39.4 ± 7 (*p* = 0.04)*
Secondary MR (n = 19)	4.48 ± 0.05 → 4.53 ± 0.08 (*p* = 0.07)	17.9 ± 0.1 → 17.0 ± 0.5 (*p* = 0.02)	9.9 ± 0.2 → 9.1 ± 0.2 (*p* = 0.03)	56 ± 9 → 48.8 ± 9 (*p* = 0.01)*
Control				
Primary MR (n = 13)	3.8 ± 0.4 → 3.9 ± 0.5 (*p*> 0.05)	18.5 ± 2.8 → 17.8 ± 3.0 (*p*> 0.05)	10.3 ± 1.4 → 9.8 ± 1.6 (*p*> 0.05)	50 ± 8 → 52 ± 8 (*p*> 0.05)
Secondary MR (n = 17)	4.5 ± 0.5 → 4.7 ± 0.6 (*p*> 0.05)	15.5 ± 2.8 → 14.3 ± 3.0 (*p*> 0.05)	9.0 ± 1.5 → 8.2 ± 1.7 (*p*> 0.05)	64 ± 9 → 66 ± 9 (*p*> 0.05)

*Values expressed as mean ± SD. * indicates statistical significance at *p* <0.05, *p-value (Group × Time)

RV systolic indices showed similar patterns. In MitraClip patients with primary MR, RV size remained stable (RV basal diameter ~ 3.5–3.7 cm at baseline and follow-up), with no significant change in TAPSE (≈ 20 mm) or RV S′ (≈ 10–11 cm/s). In secondary MR, RV basal diameter was unchanged (~ 4.5 cm), but TAPSE declined modestly (− 0.7 mm, *p* = 0.02) and RV S′ decreased significantly (− 0.8 cm/s, *p* = 0.03). These changes were mild and occurred despite reductions in SPAP.

In controls, RV function declined more substantially. TAPSE fell by an average of − 1.2 mm in secondary MR and − 0.7 mm in primary MR, while RV S′ declined by − 0.8 cm/s and − 0.5 cm/s, respectively. This resulted in nonsignificant group × time differences for both TAPSE and RV S′ (*p* > 0.05).

Overall, SPAP decreased after MitraClip (− 5 to − 7 mmHg) and increased in controls (+ 2 mmHg), with a significant overall difference (*p* ≈ 0.015). Right ventricular size and function remained stable in the MitraClip group, whereas controls showed progressive RV systolic decline and persistently elevated SPAP.

### Clinical outcomes

All patients were maintained on maximally tolerated guideline-directed medical therapy (GDMT) throughout follow-up unless contraindicated, including beta-blockers, ACEi/ARB or ARNI, mineralocorticoid receptor antagonists, diuretics, and SGLT2 inhibitors. GDMT was optimized according to tolerance and hemodynamic status, and dose adjustments were performed during follow-up visits. Selected patients also received CRT or ICD as indicated.

Table 7 shows clinical outcomes at 3-year follow-up in patients treated with MitraClip compared with controls by MR etiology.

**Table 7:** Clinical outcomes at 3-year follow-up in mitraclip and control patients

Outcome	MitraClip group				Control group			
	Total Cohort (n = 31)	Primary MR (n = 12)	Secondary MR (n = 19)	*p*-value	Total Cohort (n = 30)	Primary MR (n = 13)	Secondary MR (n = 17)	Overall group *p*-value
All-Cause Mortality, n (%)	4 (12.9%)	1 (8.3%)	3 (15.8%)	0.621	14 (45.0%)	5 (38.5%)	9 (52.9%)	0.007
HF Hospitalization, n (%)	18 (58.1%)	5 (41.7%)	13 (68.4%)	0.149	25 (83.3%)	10 (76.9%)	15 (88.2%)	0.042
Mean HF Admissions ± SD	2.1 ± 1.8	1.2 ± 1.0	2.6 ± 2.0	0.042	7.6 ± 3.2	6.8 ± 2.9	8.2 ± 3.4	< 0.001
NYHA I/II at 3 Years, n (%)	13 (41.9%)	6 (50%)	7 (36.8%)	0.487	5 (15.0%)	2 (15.4%)	3 (17.6%)	0.028
6MWT change (m, mean ± SD)	+ 20 to+ 40	+ 25 ± 10	+ 20 ± 12	0.110	− 30 ± 10	− 25 ± 12	− 35 ± 10	0.015

HF, heart failure; MR, mitral regurgitation; NYHA, New York Heart Association

**Fig. 3:**
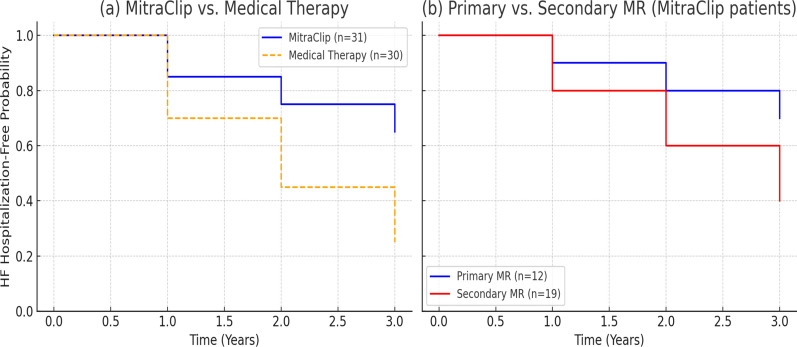
Kaplan–Meier Survival Analysis Over 3 Years **a** Survival in MitraClip patients (n = 31), compared with contemporaneous medically managed controls (n = 30). **b** Survival stratified by MR etiology among MitraClip patients. Primary MR (n = 12) compared with secondary MR (n = 19)

Over 3 years, all-cause mortality was 12.9% (n = 4), in the MitraClip cohort significantly lower compared with **45.0% (n = 14)** in controls (*p* = 0.007) at 3 years. However, Mortality did not reach statistical significance by MR etiology within MitraClip patients (8.3% in primary MR vs 15.8% in secondary MR, *p* = 0.621). Kaplan–Meier survival curves analysis (Fig. 3). But control patients with secondary MR had the highest death rates (52.9%). Causes of death in MitraClip patients included progressive HF (n = 3) and sepsis (n = 1), whereas in controls, most deaths were due to progressive HF (12/30, 40%). Of MitraClip patients treated in 2016, one remains alive with stable MR reduction.

Heart failure hospitalizations were recorded in 58.1% (n = 18), of MitraClip patients with a mean of 2.1 ± 1.8 admissions, compared with 83.3% (n = 25) of controls with a mean of 7.6 ± 3.2 admissions (*p* < 0.001). Within MitraClip, secondary MR patients had higher HF admission rates than primary MR (2.6 ± 2.0 vs. 1.2 ± 1.0, *p* = 0.042). Controls with secondary MR again showed the worst profile, averaging over 8.2 hospitalizations across follow-up.

Functional outcomes favored MitraClip. At 3 years, NYHA class I/II improvement was seen in 41.9% (n = 13) of MitraClip patients, including 50% of primary MR and 36.8% of secondary MR patients (*p* = 0.487), compared with **15%** of controls (*p* = 0.028). Similarly, 6-Minute Walk Test (6MWT***),*** the distance improved in MitraClip patients (Primary MR + 35 m; Secondary MR + 25 m), whereas controls declined (Primary MR − 25 m; Secondary MR − 35 m), yielding a significant group × time effect (*p* = 0.015). 6MWT distances at baseline and follow-up in MitraClip and control patients, stratified by MR etiology, are shown in Table 8.

**Table 8:** 6MWT in mitraclip vs. control patients by etiology

Parameter	Primary MR—MitraClip (n = 12)	Secondary MR—MitraClip (n = 19)	Primary MR—Control (n = 13)	Secondary MR—Control (n = 17)
Baseline 6MWT Range (m)	230–260	250–280	280–300	280–300
Patients with available data (%)	90%	45%	~ 80%	~ 80%
Baseline mean ± SD (m)	245 ± 9	265 ± 10	290 ± 12	288 ± 15
Baseline median [IQR] (m)	245 [238–252]	265 [257–273]	290 [285–295]	288 [280–296]
Mean change at follow-up (m)	+ 35 ± 5	+ 25 ± 5	− 25 ± 8	− 35 ± 10
Median change [IQR] (m)	+ 35 [32–38]	+ 25 [22–28]	− 25 [− 30 to − 20]	− 35 [− 40 to − 30]

6MWT, 6-minute walk test

Values are presented as mean ± standard deviation (SD) or median [interquartile range, IQR]

### BNP trends

In MitraClip patients, Median BNP levels decreased from 698 pg/mL [IQR: 263–1355] at baseline to 444 pg/mL [159–973] at 1 year, with a rebound to 608 pg/mL [228–1251] at 3 years. The baseline-to-3-years change was not statistically significant (*p* = 0.187). BNP changes had an insignificant correlation with HF hospitalizations (ρ = 0.35, *p* = 0.055), and baseline BNP levels were higher among non-survivors (median 1200 pg/mL) compared to survivors (650 pg/mL; *p* = 0.089). In contrast, control patients demonstrated persistently elevated BNP with progressive worsening over time. Median BNP was **950 pg/mL [IQR: 500–1700]** at baseline, rising to **1100 pg/mL [600–1900]** at 1 year, and peaking at **1600 pg/mL [1200–2200]** by 3 years (*p* < 0.01 for baseline-to-3-year change). Several control patients reached BNP levels > 2000 pg/mL during HF admissions.

The overall group × time interaction was significant (*p* = 0.01), confirming that BNP improvement was observed in MitraClip patients but not in controls, where BNP reflected ongoing hemodynamic burden and recurrent decompensation (Table 9).

**Table 9:** BNP analysis in mitraclip vs control patients

Timepoint	MitraClip (n = 31), median [IQR] pg/mL	Control (n = 30), median [IQR] pg/mL	p-value
Baseline	698 [263–1355] pg/mL	**950 [500–1700]**	0.12
1 st Year Follow-up	444 [159–973] pg/mL	**1100 [600–1900]**	0.031
3rd Year Follow-up	608 [228–1251] pg/mL	**1600 [1200–2200]**	0.010

IQR, Inter quartile range represents the range between the 25th and 75th percentiles, indicating the spread of the middle 50% of the data. *p*-value (<0.05) indicates statistical significance

## Discussion

This single-center, long-term follow-up study demonstrates that transcatheter mitral valve edge-to-edge repair (TEER) using the MitraClip system achieves durable reduction in mitral regurgitation (MR) severity over a 3-year period in high-surgical-risk patients. Importantly, unlike many prior single-arm studies, we included a contemporaneous medically treated control group, which strengthens the clinical relevance of our findings. The majority of mitraClip patients maintained mild-to-moderate MR or better through follow-up, with recurrence in 17% largely due to single-leaflet device attachment (SLDA), progression of leaflet pathology, or adverse left ventricular (LV) remodeling. In contrast, all control patients remained in the moderate-to-severe or severe MR range, highlighting the sustained durability of TEER compared with medical therapy alone. These findings align with prior evidence from the EVEREST and COAPT trials, which established the MitraClip as a viable and safe intervention for high-surgical-risk patients with both primary and secondary MR [6, 7].

Despite the consistent and significant reduction in MR, our durability rate was modestly lower than large multicenter registries, likely reflecting several real-world factors: predominant use of first-generation devices in the early years of the program, evolving procedural techniques and case selection strategies, and inclusion of patients with complex anatomy (12.9%) who may have been excluded from earlier trials.

Emerging evidence highlights the importance of distinguishing *proportionate* from *disproportionate* secondary MR when predicting outcomes after TEER. In proportionate MR, regurgitation severity is commensurate with LV dilation and dysfunction, and thus correction of MR often yields limited reverse remodeling and clinical benefit. In contrast, disproportionate MR refers to regurgitation severity that exceeds what would be expected from the degree of LV dysfunction, a phenotype more likely to demonstrate significant improvement after MitraClip. In our study, the majority of secondary MR cases appeared to represent proportionate MR, which may explain the absence of significant improvements in LVEF, LV dimensions, or BNP despite durable MR reduction. This observation aligns with recent studies emphasizing MR proportionality as a critical determinant of TEER benefit [12].

LV systolic function remained unchanged despite robust MR reduction, LVEF (~ 33–35%). paralleling the trajectory observed in controls. This highlights the complex interplay between MR correction and myocardial recovery. In theory, alleviating chronic volume overload should promote reverse remodeling and functional improvement. However, in practice, especially among patients with long-standing ventricular dysfunction, myocardial fibrosis, or advanced cardiomyopathy, EF recovery may be limited [13]. This was evident in our cohort, where EF remained unchanged despite significant MR improvement, suggesting that ventricular recovery is not guaranteed post-MitraClip.

Our findings mirror those of the MITRA-FR trial and differ from COAPT, where EF improvement and clinical outcomes were more notable in selected patients. The discrepancy likely results from differences in patient selection. In our cohort, a high percentage (61.3%) of patients had secondary MR, most of whom had proportionate MR with advanced LV remodeling and permanent myocardial damage. By contrast, primary MR patients, whose pathology was primarily valvular, demonstrated greater MR reduction, functional gains, and stability of RV function, albeit still with limited reverse remodeling. Our MR reduction rate (83.9%) was comparable to COAPT (82%) and higher than MITRA-FR (59%), suggesting appropriate patient selection. However, the HF hospitalization rate (58.1%) was considerably higher than COAPT (35.7%), likely due to the higher proportion of secondary MR cases compared to primary MR (68.4% vs. 41.7%) in our cohort and advanced ventricular dysfunction despite optimized GDMT. This underscores the persistent burden of ventricular dysfunction despite MR correction [7, 14]. Propensity-adjusted analyses further supported the association between primary MR and more favorable outcomes, in line with EVEREST II [6].

Pulmonary and right-sided hemodynamics, however, diverged significantly between groups. SPAP declined after MitraClip (− 5 to − 7 mmHg) but rose modestly in controls, with a significant group × time effect (p ≈ 0.015). RV systolic function (TAPSE and RV S′) was largely preserved in MitraClip patients but declined in controls, suggesting that TEER stabilizes RV performance and prevents progressive deterioration, particularly in primary MR. These findings are consistent with published reports showing that while TEER may not fully reverse RV remodeling, it can mitigate worsening right-sided function [15, 16].

Biomarker and functional data further reinforce the benefit of MitraClip. BNP levels decreased after TEER before modest rebound at 3 years, while controls demonstrated sustained rises with peaks > 2000 pg/mL in decompensated patients. The overall group × time effect was significant (*p* = 0.01), confirming a biomarker advantage with TEER. Functional capacity mirrored this trend: MitraClip patients gained + 25 to + 35 m in the 6-min walk test (6MWT), whereas controls declined by − 25 to − 35 m, with a significant group × time interaction. NYHA class improved in 41.9% of MitraClip patients compared with only 15% of controls. Together, these findings provide objective and subjective evidence of functional benefit after MitraClip. However, absence of systematic quality-of-life (QoL) assessments, such as Kansas City Cardiomyopathy Questionnaire (KCCQ) scores, remains a limitation.

Procedural safety was favorable, with no in-hospital mortality and a low incidence of complications. Transmitral gradients increased modestly but remained within physiologic range, with only one patient developing moderate post-procedural mitral stenosis after double-clip implantation. Anatomical suitability was assessed per EVEREST criteria, although some patients had challenging morphologies (e.g., restricted leaflet mobility, calcification) requiring procedural adaptation. Early in the study, most cases were performed with first-generation NT devices, which had narrower clip arms and limited grasping compared with later NTW or G4 systems. These technical factors likely contributed to the modestly lower MR durability observed compared with contemporary multicenter registries.

Additionally, echocardiographic data showed only modest decreases in LVEDV, LVESV, and linear dimensions (LVEDD and LVESD), further suggesting limited reverse remodeling. This lack of significant structural change aligns with persistent myocardial dysfunction and may partly explain the unchanged EF. In chronic cardiomyopathy, structural alterations such as fibrosis may be irreversible, decreasing the heart’s ability to recover even after volume unloading. These observations emphasize the importance of early intervention in primary MR patients who are high surgical risk but still have preserved ventricular function. Conversely, in secondary MR, careful patient selection, comprehensive HF management, and realistic outcome expectations are crucial. Future studies should investigate combination strategies that integrate MitraClip with newer pharmacologic or device-based therapies to improve outcomes. These findings also advocate for a holistic management strategy, combining valve repair with heart failure therapies targeting neurohormonal and structural pathways [17, 18].

Importantly, this study contributes novel regional data, presenting long-term (3-year) echocardiographic and clinical outcomes from a Middle Eastern population, a group underrepresented in landmark trials. These findings extend the external validity of TEER benefits across diverse healthcare settings and patient demographics. Future prospective multicenter studies with larger and more diverse cohorts**,** standardized echocardiographic protocols**,** and robust clinical endpoints are warranted to validate and expand upon these findings, particularly in underrepresented populations and real-world settings [6–8, 16–19].

### Limitations

This study has several limitations. First, the small sample size (n = 31) limits statistical power, particularly for subgroup analysis and restricts generalizability. Second, the single-center, retrospective design introduces potential referral and selection bias, and results should therefore be interpreted as exploratory and hypothesis-generating, pending validation in larger multicenter studies.. Third, Echocardiographic assessments lacked core-lab adjudication; only LVEF discrepancies were resolved by averaging independent measurements, while MR parameters were adjudicated by consensus, which may introduce bias. Fourth, Quality-of-life measures such as KCCQ were not available, and functional capacity (6MWT) and BNP data were incomplete in few patients. Finally, early use of first-generation MitraClip devices may have contributed to lower durability compared with newer-generation systems.

## Conclusion

In this study, TEER with the MitraClip system provided durable and significant MR reduction, survival benefit, and improved functional outcomes over 3 years in high surgical-risk patients, with clear superiority over medical therapy alone. The most pronounced benefits were seen in primary MR, where better baseline ventricular function supported sustained MR reduction and improved clinical status. In contrast, patients with secondary MR derived less functional recovery and experienced higher rates of HF hospitalization, underscoring the persistent burden of ventricular disease and the need for optimized guideline-directed therapy.

These findings emphasize the importance of distinguishing proportionate from disproportionate secondary MR, prioritizing early referral before advanced remodeling, and embedding TEER into a comprehensive, multidisciplinary HF management strategy. While our results extend the evidence base by including a Middle Eastern cohort, the small sample size, single-center retrospective design, and predominant use of early-generation devices warrant cautious interpretation. Larger, prospective multicenter studies with contemporary devices, standardized imaging, quality-of-life assessments, and systematic control groups are needed to validate and expand upon these observations.

## Data Availability

No datasets were generated or analysed during the current study.

## Abbreviations

BNP: B-type natriuretic peptide
EROA: Effective regurgitant orifice area
HF: Heart failure
LVEDD: Left ventricular end-diastolic diameter
LVEDV: Left ventricular end-diastolic volume
LVESD: Left ventricular end-systolic diameter
LVESV: Left ventricular end-systolic volume
LVEF: Left ventricular ejection fraction
MR: Mitral regurgitation
NYHA: New York Heart Association
RF: Regurgitant fraction
RVol: Regurgitant volume
SD: Standard deviation
TEER: Transcatheter edge to edge repair
6MWT: Six-minute walk test
